# Cranial Nerve Enhancement in Multiple Sclerosis Is Associated With Younger Age at Onset and More Severe Disease

**DOI:** 10.3389/fneur.2019.01085

**Published:** 2019-11-06

**Authors:** Lukas Haider, Wei-Shin Evelyn Chan, Elisabeth Olbert, Stephanie Mangesius, Assunta Dal-Bianco, Fritz Leutmezer, Daniela Prayer, Majda Thurnher

**Affiliations:** ^1^NMR Research Unit, Department of Neuroinflammation, Faculty of Brain Science, Queen Square MS Centre, UCL Institute of Neurology, University College London, London, United Kingdom; ^2^Department of Biomedical Imaging and Image-Guided Therapy, Medical University Vienna, Vienna, Austria; ^3^Department of Neurology, University Hospital Tulln, Tulln, Austria; ^4^Department of Neuroradiology, Medical University of Innsbruck, AUT, Innsbruck, Austria; ^5^Neuroimaging Core Facility, Medical University of Innsbruck, AUT, Innsbruck, Austria; ^6^Department of Neurology, Medical University Vienna, Vienna, Austria

**Keywords:** multiple sclerosis, magnetic resonance imaging, contrast media, cranial nerves, brain stem, retrograde neurodegeneration

## Abstract

**Background:** The overall frequency of cranial nerve pathology, including cranial nerves other than the trigeminal nerve, as well as its relation to brainstem lesion formation on magnetic resonance imaging (MRI) and clinical correlates in multiple sclerosis (MS) is unknown.

**Objective:** We aimed to determine the frequency of cranial nerve enhancement on MRI, and its association with brainstem lesion formation and clinical outcomes.

**Methods:** We retrospectively analyzed, in 183 patients, (RRMS: 156, SPMS: 15, PPMS: 6, CIS: 6) 651 MRIs (76.5% on the identical scanner Siemens Trio Tim, 3T with identical MRI protocols). Frequencies of cranial nerve enhancement on post contrast T1-weighted MRIs were compared to lesion counts and the MS-severity-score.

**Results:** Cranial nerve enhancement was present in 8.2% of the analyzed MS patients (oculomotor-nerve: 1.1%, trigeminal-nerve: 2.7%, abducens-nerve: 2.2%, facial-/vestibulocochlear nerve: 1.6%, vagal-nerve: 0.5%). Of those, 13% suffered from repeated episodes and 27% exhibited a cranial nerve enhancement duration of >12 months. Age at MS onset was lower in patients with cranial nerve enhancement, 23 vs. 28 years, *p* = 0.049. The MS-severity-score, 5.15 vs. 0.88 (*p* = 0.019), the T2 brainstem-, 1 vs. 0 (*p* = 0.041), and the total intracranial contrast-enhancing lesion counts, 2 vs. 0 (*p* = 0.000), were higher in patients with cranial nerve enhancement, compared to age-, disease duration-, and gender- matched MS patients.

**Conclusions:** Cranial nerve enhancement, present in 8.2% of our patients, was associated with a younger age at MS onset, brainstem lesions, and a more severe disease course.

## Introduction

Multiple Sclerosis (MS) is a chronic inflammatory disease of the central nervous system (CNS) (1). Isolated cranial nerve (CN) involvement was clinically reported in 10.4% (50/483) of all MS patients observed during a 5-year period, with 7.3% of those in whom CN involvement was a presenting symptom of MS, and, in 3.1%, as a sign of disease relapse (2). The oculomotor nerve was found to be clinically affected in 2%, the trigeminal nerve in 23%, the abducens nerve in 5%, the facial nerve in 18% and the vestibulocochlear nerve in 2% (2). This is in contrast to magnetic resonance imaging (MRI) studies, where systematically assessed data has thus far reported involvement of the trigeminal nerve only. On post contrast T1-weighted MRI, 2.8% (24/851) of MS patients exhibited enhancement of the cisternal portion of the trigeminal nerve at 0.5–1.5 Tesla (T) (3) and 2.9% (8/275) of patients at 1.0 T (4). By using 3D T2 turbo spin echo, 3D T2 fluid attenuated inversion recovery and 3D T1 inversion recovery and up to 0.5 mm in-plane resolution at 3 T, 23% (11/47) of patients displayed signal abnormalities in the central trigeminal pathways, including the root entry zone and either the trans-cisternal nerve or pontine nucleus (5). The possibility to visualize cisternal or central involvement of other (smaller) cranial nerves on MRI examinations has been reported in three case reports (6–8). Uzawa et al. reported an abducens palsy and facial nerve palsy due to a facial collicular plaque (6). Two cases were reported with clinical and radiological oculomotor nerve involvement in association with cerebral peduncular lesions close to the emergence of the third nerve and continuity of the enhancement between the demyelinated lesion and the cisternal portion of the third nerve (7, 8). While detection of cranial nerve enhancement (CNE) in cranial nerves other than the trigeminal nerve on 3T MRI performed for clinical purposes, is indicated, its frequency and its relation to brainstem lesion formation and clinical parameters is remains unknown. In this retrospective study, we therefore aimed to determine: (a) the frequency of cranial nerve enhancement (CNE) in randomly selected MS patients; (b) the dynamic on follow-up examinations; and (c) its correlation with disease severity and age at onset.

## Materials and Methods

### Study Design

We selected 183 MS patients between 2002 and 2017 through our hospital information system (SAP SE) using the following selection criteria: (a) data entry via the MS-Neurology-Outpatient-Department; and (b) availability of more than one brain MRI.

From a total of 838 MRI examinations 120 were excluded due to lack of pre- and post-contrast T1-weighted images (WI), 58 MRI examinations were excluded due to image artifacts (e.g., patient motion-related), or low resolution. A total of 651 MRI exams in 183 patients were included in the study (Table 1). A median of three MRI examinations (2–4) were analyzed per patient. The median time interval between baseline and follow-up MRIs was 834 days (336–1,850 days; 25–75 percentile range). There were 234 MRI examinations (36%) performed on a 1.5 T MRI clinical scanner, and 417 (64%) on a 3.0 T clinical scanner (Siemens Trio Tim).

**Table 1:** 183 MS patients, with more than one available brain MRI, were randomly selected between 03.04.2002 and 03.11.2017, for a total of 829 MRI examinations.

**Inclusion and exclusion of cMRI studies**	***n***
Randomly selected MS patients with >1 cMRI examination	183
cMRI examinations in total	829
Excluded cMRI (due to)	−178
lack of pre- or post contrast T1-WI	−120
low spatial resolution or artifacts (e.g., motion artifacts)	−58
Analyzed cMRI	651

*From these, 178 MRI examinations were excluded due to lack of pre or post contrast T1-weighted images (120 examinations) and low resolution or motion artifacts (58 examinations). In total, 651 MRI examinations were analyzed in 183 MS patients, median 3 (2–4) per patient. cMRI, cerebral MRI; n, number*.

### Participants

We included individuals with the diagnosis of clinically definite multiple sclerosis according to the McDonald criteria (9) (RRMS: 156, SPMS: 15, PPMS: 6) and 6 individuals with the diagnosis of clinically isolated syndrome. The clinical data assessed included date of birth, gender, disease onset, disease course, Expanded Disability Status Scale (EDSS), and cranial nerve involvement (Table 2). The MS severity score (MSSS) was calculated according to Roxburgh et al. (10). Lumbar puncture was routinely performed in all MS patients in the course of the initial diagnostic process to rule out infectious diseases, especially viral or borrelia-induced neuritis. We did not collect data on MS specific treatments. The clinical annotations were derived from retrospective review of clinical routine charts.

**Table 2:** Participants were categorized intro clinical groups according to the McDonald criteria (9), (RRMS: 156, SPMS: 15, PPMS: 6, CIS: 6).

**Disease type**	**Age (at MS onset)**	**Age (at BL MRI)**	**Age (at last MRI)**	**Female/ male**
CIS	31 (27–39)	30 (27–37)	31.5 (28–38)	6/0
RRMS	27 (20–36)	32 (22–39)	34 (26.5–43)	104/52
SPMS	31.5 (23–42)	46 (41–51)	49 (44–58)	11/4
PPMS	31 (28–33)	35 (32–38)	43 (35–44)	3/3

*Gender, age, age at MRI and disease duration were available for the entire cohort*.

### Subgroup Selection

Two randomly selected groups of MS patients without CNE were matched for both gender and disease course. One group was also age-matched (*n* = 15), whereas the other group was matched for age at disease onset (*n* = 15).

### Image Acquisition

There were 76.5% (319/417) examinations performed on a clinical Siemens Trio Tim scanner (3T) with a TR (repetition time) of 1800 ms, a TE (time to echo) of 2.19–3.79 ms, a TI (inversion recovery) of 900–1,100 ms, a matrix of 192 × 192–484 × 484, and a slice thickness of 1 mm (Supplementary Table 1). The contrast agents Gadobutrol and Gadoteric acid were intravenously applied at a dose of 0.1–0.2 ml/kg. Due to the retrospective design we could not control for the time intervals from injection to post-contrast MRI. Scans from other MRI machines, including 1.5 T were included in the initial analysis, however, they did not contribute to the total number of cranial nerves with enhancement, which was only measured on 3Tscans (see section Results). We allowed for change between MRI scanners, e.g., baseline at 3T, FU-1 at 1.5, FU-2 at 3T.

### Image Analysis

CN enhancement on post-contrast T1-(WI) in the cisternal segment was determined by consensus rating performed by three trained readers (WSEC, TZ, and LH). In the case of disagreement, expert rating was considered (MT). The optic nerve was excluded from the analysis because of its diencephalic origin and oligodendrocyte myelination. Cases of suspected CNE based on this initial review were subsequently subjected to region-of-interest (ROI)-based manual measurement of the CN signal intensity (SI) on pre- and post-contrast T1 WI with IMPAX EE R20 XV SU3, AGFA Healthcare, Mortsel, Belgium using multiplanar and curved reconstructions along the course of the cranial nerve. Therefore, a freehand ROI was drawn around each CN with suspected enhancement, using similar distances between the cerebrospinal fluid (CSF) and the CN in its cisternal segment on pre- and post-contrast T1 WI. Each SI was normalized by the SI of the CSF in the lateral ventricles of the same MR sequence. The ratio between the CSF-normalized CN-Sis on pre- and post-contrast images was calculated. CNs with an increase of signal intensity >110% was ultimately rated as positive. In these MS patients, the SI of the affected CN was also measured in the same manner in a second, negatively rated MR scan (Supplementary Figure 1) to prove the absence of an SI increase in negatively rated MRI scans. In two of all the cases with CNE, the affected CN was rated positively in all available MR scans.

Brainstem lesion load was manually counted on T2-weighted images (T2-WI). The total number of intracranial-enhancing lesions was determined on post-contrast T1-weighted images.

### Statistical Analysis

The descriptive statistical analyses were performed using IBM SPSS Statistics 21 (IBM, Armonk, NY, USA). Categorical variables are presented as number (percentage), numerical variables are shown as median (25–75 percentile range). Group differences were calculated using the Mann–Whitney *U*-test.

## Results

**Figure 1:**
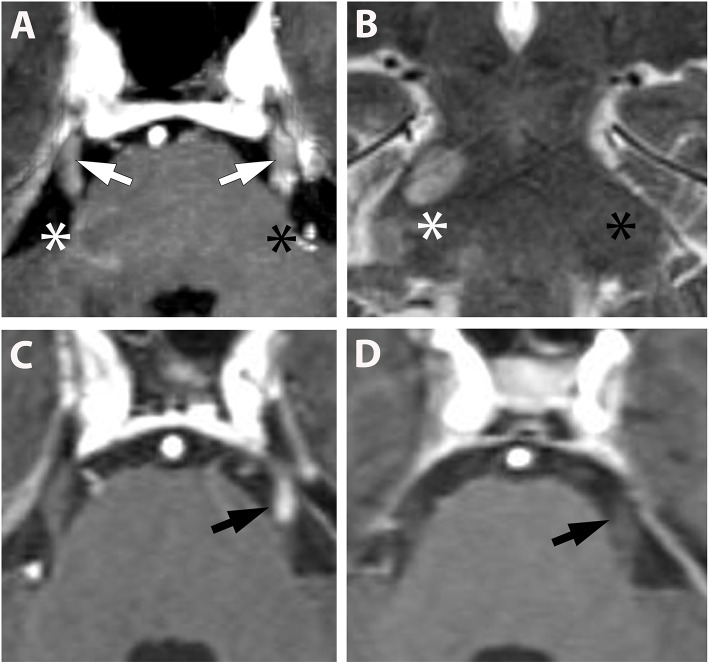
**(A,B)** (Pat. No. 2): Axial T1-weighted MRI post contrast enhancement **(A)** at the level of the pons and a corresponding coronal T2-weighted MRI of the same patient **(B)**. On post contrast images, intense bilateral contrast enhancement is depicted in the cisternal segment of the trigeminal nerve (white arrows). A ring-enhancing lesion is located at the root entry zone of the right trigeminal nerve (white asterisk) **(A)**. No root entry zone lesion is visualized at the contralateral site (black asterisk) **(A,B)**. **(C,D)** (Pat. No. 11): Axial T1-weighted MRIs post contrast enhancement **(A,B)** at the level of the pons, at baseline **(C)**, and at follow-up MRI 11 months later **(D)**.

**Figure 2:**
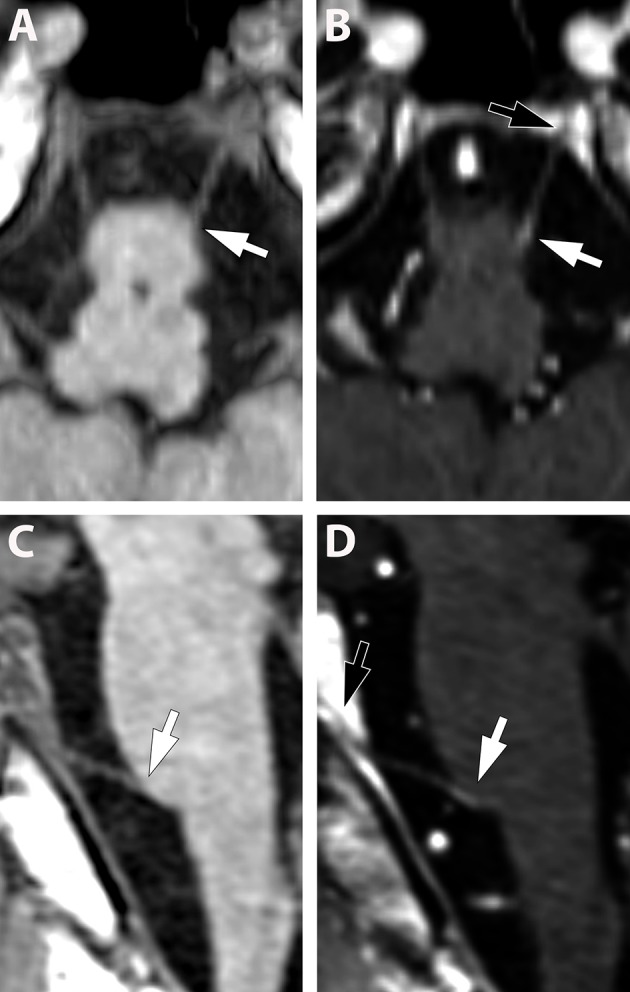
**(A–D)** (Pat. No. 10): Pre-contrast **(A,C)** and post contrast enhancement **(B,D)**, axial **(A,B)**, and sagittal **(C,D)**, reconstructed T1-weighted MRIs at the level of the abducens nerve. Due to the small diameter of the cranial nerves, other than the fifth nerve, the contrast enhancement is difficult to detect (white arrows in **B,D**) and multi-planar reconstructions are helpful. Identification of the abducens nerve and differentiation from blood vessels can be facilitated by visualization of the abducens nerve in Dore'lo's canal (black arrow **B,D**).

Overall, cranial nerve enhancement (CNE) was present in 8.2% (15/183) of the analyzed MS patients. Trigeminal nerve enhancement was detected in 2.7% (5/183), oculomotor nerve enhancement was present in 1.1% (2/183), abducens nerve enhancement in 2.2% (4/183), vestibulocochlear or facial nerve enhancement in 1.6% (3/183), and vagus nerve enhancement in 0.5% (1/183) (Table 3). No contrast enhancement was observed in olfactory, trochlear, glossopharyngeal, accessory, or hypoglossal nerves. CNE was bilateral in 20% (3/15), left-sided in 40% (6/15), right-sided in 20% (3/15), and bilateral but left-dominated in 20% (3/15) (Table 3, Figures 1A,C). CNE was found either proximally (Figures 2B,D, when compared to the pre-contrast images, Figures 2A,C), distally, or along the entire cisternal segment of the CN (Figures 1A,C).

**Table 3:** Oculomotor nerve (III) enhancement was detected in 1.1% (2/15), trigeminal nerve (V) enhancement was detected in 2.7% (5/15), abducens nerve (VI) enhancement in 2.2% (4/15), vestibulocochlear or facial nerve (VII/VIII) enhancement in 1.6% (3/15), and vagal nerve (X) enhancement in 0.5% (1/15).

**Pat.No**.	**CN involved**	**Side of CNE**	**Duration of CNE**	**REZ involvement on T2**	**Lesion counts**		**Number of analyzed MRIs**	**MRI Follow-up [months]**
					**T2; Brainstem**	**CEL; Total brain**		
12	III	Bilateral	NDA	No	1	1	2	10.8
14	III	Left	NDA	Yes	1	2	2	2.4
2	V	bilateral	>5 months	Right: yes, left: no	1	5	2	0.6
7	V	Left>right	<98 days	Left: yes	2	19	4	14.6
8	V	Left>right	>20 months	No	0	0	4	115.1
11	V	Left	<11 months	No	1	0	2	10.8
15	V	Left	>15 months	No	0	1	2	15.1
3	VI	Bilateral	NDA	No	1	1	2	104.2
6	VI	Left>right	>21 months	No	2	3	4	21.6
10	VI	Left	1st: <12 months; 2nd: <22 months	No	0	2	10	102.1
13	VI	Right	>13 months	No	0	3	3	42.1
1	VII/VIII	Left	1st: <18 days; 2nd: <12 months	Yes	3	20	16	106.4
5	VII/VIII	Left	<20 months	No	0	0	2	19.8
9	VII/VIII	Right	NDA	No	1	5	2	1.8
4	X	Right	NDA	Yes	4	7	3	28.5

*CNE was bilateral in 20% (3/15), left-sided in 40% (6/15), right-sided in 20% (3/15), and bilateral but left-dominated in 20% (3/15). Estimations for the duration of CNE were not available for 33% (5/15) because CNE was present only in the last available MRI scan in these individuals. A CNE duration of >5 months was present in 7% (1/15), and a duration of >12 months in 27% (4/15). Patient No. 10 and No. 1 displayed two episodes of CNE on follow-up MRI. In patients with CNE, MS plaques near the root entry zone (REZ) of the affected CN were depicted on T2 or FLAIR images in 33% (5/15) of MS patients. In patients with CNE, a median of one lesion (0–2) was detected in the brainstem (mesencephalon, pons, and medulla oblongata) and two (1–5) contrast-enhancing lesions (CEL) in the total neurocranium. In patients with CNE, two MRIs were analyzed in 53% (8/15), three MRIs in 13% (2/15), four MRIs in 20% (3/15), ten MRIs and 16 MRIs each in 7% (1/15). The median follow-up period for individuals with CNE spanned 19.8 months (10.8–102.1)*.

**Figure 3:**
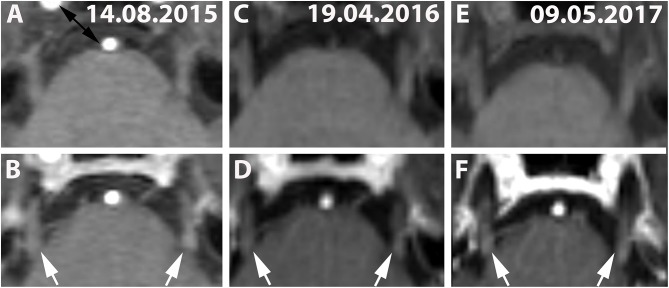
**(A,C,E)** (Patient No. 8): Pre-contrast and corresponding post contrast T1-weighted axial images **(B,D,F)** reveal long-lasting bilateral (left-dominated) trigeminal enhancement (white arrows) of >20 months on three follow-up MRIs (**A,B**: 14.08.2015; **C,D**: 19.04.2016; **E,F**: 09.05.2017). No brainstem lesion was detected in this case on T2-weighted images (not shown).

Estimations for the duration of CNE were not available for 33% (5/15) because CNE was present only in the last available MRI scan in these individuals. Although CNE was found to be a temporarily limited finding in 7% (1/15) (Figures 1C,D), it lasted for >5 months in 7% (1/15) and >12 months in 27% (4/15) (Table 3 and Figure 3). Patients No. 10 and No. 1 displayed two episodes of CNE on follow-up MRIs.

T2 lesions with extension to the proximity of the root entry/exit zone (REZ) were detected in 33% (5/15) of MS patients (Figures 1A,B, white asterisk). Contrast enhancement of such REZ lesions showed continuous extension into the enhancing CN (Figure 1A white asterisk). In one of these five patients the MS lesion in the REZ preceded the CNE (by 18 days). We did not observe a case were CNE preceded REZ lesion formation. Overall, patients with CNE had a median of one T2 lesion (0-2) in the brainstem and two contrast-enhancing lesions (CEL) (1–5) in the total neurocranium (Table 3). Two randomly selected groups of MS patients without CNE were matched for both gender and disease course. One group was also age-matched, whereas the other group was matched for age at disease onset. Compared to patients with CNE, there was a significant lower frequency of T2 brainstem lesions *n* = 0 (0–0); *p* = 0.041 in age-matched MS patients. This difference in T2 brainstem lesions was lost when patients were matched for age at disease onset [*n* = 1 (0–1), *p* = 0.267] (Supplementary Table 2). CEL counts were significantly lower in the MS group without CNE matched for age [*n* = 0 (0–0); *p* = 0.000] and age at MS-onset [*n* = 0 (0–0); *p* = 0.001].

Twenty-seven percent (4/15) of the patients with CNE had a juvenile disease onset before the age of 18 years and a significantly lower age at MS onset of 23 years (15–29 years), compared to patients without CNE with MS onset at 28 years (22–36 years), *p* = 0.049. MS severity score (MSSS) values (Table 4) were significantly higher in patients with CNE [MSSS 5.15 (1.89–6.14)] compared to the control groups, matched for age [MSSS 0.88 (0.35–3.19); *p* = 0.019] and age at MS-onset [MSSS 1.04 (0.64–3.69); *p* = 0.033].

**Table 4:** A clinical record compatible with CN involvement was found in 33% (5/15) by retrospective review of routine neurological examinations.

**Pat. No**.	**CN involved**	**Clinical record of CNE**	**Disease type**	**M/F**	**Age at MS onset [years]**	**Disease duration at incidence of CNE [days]**	**MS severity score (MSSS)**
12	III	No	RRMS	F	20	327	2.44
14	III	Yes	RRMS	M	29	72	0.67
2	V	No	RRMS	M	42	1	9.88
7	V	No	RRMS	F	14	804	0.35
8	V	No	RRMS -> SPMS	F	19	8,469	7.38
11	V	Yes	RRMS	F	23	6,138	1.89
15	V	No	RRMS	M	15	3,970	4.94
3	VI	No	RRMS	F	14	3,276	0.21
6	VI	No	CIS -> RRMS	F	38	1	6.0
10	VI	No	RRMS -> SPMS	M	27	4,917	2.34
13	VI	No	RRMS	F	20	7,526	5.15
1	VII/VIII	Yes	RRMS	M	13	66	6.14
5	VII/VIII	Yes	RRMS	F	30	1,552	5.79
9	VII/VIII	No	RRMS	M	28	39	5.41
4	X	Yes	RRMS	M	24	2,388	9.33

*Between the first and last MRI, 7% (1/15) changed from clinically isolated syndrome (CIS) to relapsing remitting MS (RRMS) and 13% (2/15) changed from RRMS to secondary progressive MS (SPMS); all other cases remained RRMS. The female/male ratio was 8/7. There were 27% (4/15) who had a juvenile disease onset before the age of 18. The median age of MS-onset was 23 (15–29). In 40% (6/15), CNE presented within the first year after disease onset, and 13% (2/15) had CNE on their initial MRI at disease start. Patients with CNE had a median MS severity score (MSSS) of 5.15 (1.89–6.14) (25–75% range). Patient No. 2 suffered from intravenous drug abuse; Patient No. 1 presented with a juvenile Marburg-type MS*.

Isolated CN palsy or neuralgia, at the time of, or any time after, CNE on MRIs, was found via retrospective chart review in 33% (5/15) of all patients with CNE. In 40% (6/15), CNE presented within the first year after disease onset, and 13% (2/15) had CNE on their initial MRI at disease start.

The total cohort included 156 patients with RRMS, 15 patients with SPMS, 6 patients with PPMS and 6 patients CIS. During the follow-up interval 1 patient changed from CIS to RRMS and 10 patients changed from RRMS to SPMS. In the cohort with CNE, seven percent (1/15) of patients with CNE changed from clinically isolated syndrome (CIS) to relapsing remitting multiple sclerosiss (RRMS) and 13% (2/15) changed from RRMS to secondary progressive multiple sclerosis (SPMS), whereas all other subjects remained RRMS throughout the median follow-up period of 19.8 months (10.8–102.1). The distribution of the disease courses did not reach statistical significance between patients with and without CNE (*p* = 0.353). Neither did female/male ratios differ significantly (*p* = 0.213) between cases with (8/7) and without CNE (116/52) (Table 4).

## Discussion

Cranial nerve enhancement (CNE) was present in 8.2% (15/183) of our randomly selected MS patients, with a median of three scans/patient (in total, 651 scans) analyzed at a median follow-up interval of 19.8 months. The optic nerve was excluded from the analysis because of its diencephalic origin and oligodendrocyte myelination (11). Due to the small diameter of the cranial nerves, other than the fifth nerve, the contrast enhancement is challenging to detect and multiplanar reconstructions are helpful. Oculomotor nerve enhancement was present in 1.1% (2/183), trigeminal nerve enhancement in 2.7% (5/183), abducens nerve enhancement in 2.2% (4/183), vestibulocochlear or facial nerve enhancement in 1.6% (3/183), and vagus nerve enhancement in 0.5% (1/183) (Table 3). While involvement of CNs other than the trigeminal nerve has been reported by only three case reports—one on the abducens nerve (6) and two on the oculomotor nerve (7, 8)—similar frequencies of peripheral trigeminal nerve involvement on MRI have been reported previously (2.8–2.9%), despite only one scan per patient analyzed at 0.5-1.5 T (3, 4).

This might be explained by recurrent CNE, which was found in 13% (2/15), by long-lasting CNE of >5 months in 7% (1/15) and by CNE >12 months, present in 27% (4/15), despite exclusion of so-called “normal facial nerve enhancement” (12). To our knowledge, we provide the first systematic description of longitudinal changes of CNE (Table 3 and Figure 3). Anterograde trans-synaptic degeneration has been established as a mechanism to explain optic neuritis related white matter abnormalities in the optic radiation in MS (13). In this model, changes in the posterior visual pathways relate to primary optic nerve defects in the connected tract system (14). Glial activation and demyelination in the posterior visual pathways precede the appearance of disrupted axonal transport (14). We hypothesize, that CNE, as phenomenologically observed in our study (15), might be a consequence of such tract-related myelin disintegration in the course of ante/retrograde/ trans-synaptic neurodegeneration (16, 17). In the setting of multiple sclerosis it would follow both white matter lesions (18) and diffuse axonal injury (19). Due to the nature of MS lesion formation, features such as repeated, long lasting and bilateral CNE could be explained by axonal injury in the course of lesion formation along the connected nuclear tract system of the cranial nerves (18). This view is further supported by the observation that contrast enhancement of REZ lesions frequently showed continuous extension into the enhancing CN (Figure 1A white asterisk). In one of these five patients, the MS lesion in the REZ preceded the CNE (by 18 days) and we did not observe a case were CNE preceded REZ lesion formation. The slow spread of neurodegeneration (up to weeks-months) and the lack of a clear/unique hierarchical neuronal architecture in cranial nerves (as it is inherent to e.g., the visual pathway) will, however, make the hypothesis of central neurodegeneration mediated enhancement of peripheral cranial nerves challenging to validate.

In line with a previous report of central trigeminal involvement in MS patients of 23% (5), we observed direct extension of brainstem lesions in close proximity to the CN-REZ in 33% (5/15) of patients with CNE. Patients with CNE revealed significantly more [*n* = 1 (0–2)] T2 brainstem lesions than patients matched for age, gender, disease duration, and disease course [*n* = 0 (0–0); *p* = 0.041]. This difference was lost when patients were matched for age at MS onset [*n* = 1 (0–1); *p* = 0.267) (Table 3 and Supplementary Table 2).

Although glossopharyngeal neuralgia has been described as a rare finding in MS (20), and vocal cord paralysis is rare in patients with MS (21–23), patient No. 4 represents, to our knowledge, the first reported case suggestive of a brainstem lesion associated with vagus nerve involvement in MS. This patient presented with a newly detected, subtle right-sided cisternal enhancement of the vagal nerve and a non-enhancing lesion in its REZ (Supplementary Figure 2). Contralateral uvular deviation and ipsilateral palatal drop were noted in a clinical examination, performed 6 days after the MR exam, which was clinically compatible with involvement of the right vagal nerve (Table 3).

A possible clinical correlate for CNE was present in 33% (5/15) at the time of, or after CNE, affecting the oculomotor, trigeminal, facial/vestibulocochlear, and vagal nerves (Table 4). The clinical annotations were derived from a retrospective review of clinical routine charts. The examination of cranial nerves is a standard part of the clinical routine examination. Although the routine setting might lower our sensitivity to detect a clinical correlate of CNE (e.g., due to different clinical raters), it is unlikely to increase it.

While the significant predominance of secondary progressive multiple sclerosis (SPMS) cases in patients with trigeminal enhancement has been reported (4), this finding was not present in our cohort (*p* > 0.5) (Table 4). CNE manifested in 40% (6/15) within the first year after disease onset (Table 4). In patients with CNE enhancement, 27% (4/15) had a juvenile disease onset before the age of 18 years and the median age in this group of 23 years (15–29 years) was significantly lower than the median age of patients without CNE at 28 years of age (22–36 years), *p* = 0.049. Patients with pediatric-onset MS have been previously reported to suffer from more and larger T2 lesions than adults on baseline and follow-up MRIs (24), and a higher frequency of brainstem attacks (25). In line with these findings, patients with CNE revealed more brainstem lesions [median 1 (0–2)] and higher MSSS [5.15 (1.89–6.14)] than age-matched MS patients [0(0–0); *p* = 0.41 and 0.88 (0.35–3.19); *p* = 0.019, respectively], despite the inherent limitations of retrospective study design and small sample size.

CNE of the fifth cranial nerve has been reported previously at 1.5T at a frequency of 2.8% (4) and we therefore included 36% (234/651) of 1.5T scans in our analysis. However, all patients with CNE in our study, 8.2% (15/183), were detected on 3T MRI, suggesting an increase in the detection rate with higher field strength. In 76.5% (319/417) of all cases analyzed on 3T images, MR protocol parameters were standardized (Supplementary Table 1). Due to the retrospective study design, we could not control for potentially important factors, known to influence contrast enhancement on MRI, such as the timespan between contrast application and image acquisition and the exact T1 sequence parameters and gadolinium concentration/patient for all scans. Additionally, patients in a tertiary MS center might more often be in an active inflammatory stage of disease and receive MRIs more frequently than patients with long or benign disease courses (26). We could not control the MS relapse to MRI intervals and MS treatments prior to MRI. We, therefore, suggest that our data on CNE cannot be generalized to an overall CNE prevalence in MS, but rather reflect a scanner-, MRI protocol-, and detection threshold-dependent prevalence of CNE, in a cohort that is routinely imaged in an outpatient department setting. Clinical annotation was derived from retrospective chart review. These data were not recorded in a standardized fashion and, thus, could not generate a standardized, quantitative, comprehensive dataset.

This study is further limited by the lack of a control/ reference tissue, which is not available from a biological point of view and the lack of a control patient cohort, which is not available due to restrictive and indication determined use of contrast agents in our department. The analysis was further restricted to a homogenous MS cohort and does not include other chronic CNS conditions, such as Neuromyelitis Optica Spectrum Disorders (NMOSD).

Taken together, our data indicate neurodegeneration via brainstem lesion formation as a potential source for CNE. The qualitative and quantitative detection threshold of CNE by MRI is reached more easily at higher field strengths, in patients with more aggressive MS disease courses and higher brainstem lesion loads. Tailored MR protocols and dedicated image analysis might significantly increase visualization of clinically relevant and potentially underestimated cranial nerve enhancement in patients with multiple sclerosis.

## Data Availability Statement

The datasets for this manuscript are not publicly available because data privacy can not be ensured due to potential facial recognition on the cranial MRIs used for this study. Requests regarding data access should be directed to: LH, lukas.haider@meduniwien.ac.at; MT, majda.thurnher@meduniwien.ac.at.

## Ethics Statement

The studies involving human participants were reviewed and approved by 1464/2017. Written informed consent for participation was not required for this study in accordance with the national legislation and the institutional requirements.

## Author Contributions

LH, W-SC, EO, SM, AD-B, FL, DP, and MT contributed to the study design, data acquisition, data analysis, data interpretation, manuscript drafting, and approved the final version.

### Conflict of Interest

The authors declare that the research was conducted in the absence of any commercial or financial relationships that could be construed as a potential conflict of interest.

## Abbreviations

CN: cranial nerve
CNE: cranial nerve enhancement
CNS: central nervous system
MRI: magnetic resonance imaging
MS: multiple sclerosis
MSSS: multiple sclerosis severity score
NDA: no data available
REZ: route entry/exit zone
ROI: region of interest
SI: signal intensity
T: Tesla
T1-WI: T1-weighted images
T2-WI: T2-weighted images
TE: time to echo
TI: inversion recovery
TR: repetition time.
