# Supplementary material for: Cranial Nerve Enhancement in Multiple Sclerosis Is Associated With Younger Age at Onset and More Severe Disease
Source: Front Neurol. 2019 Nov 6;10:1085. doi: 10.3389/fneur.2019.01085 (PMC6851051; doi:10.3389/fneur.2019.01085)
Supplement: Supplementary file 1 [file Data_Sheet_1.docx]

**Supplementary Table 1:**

| **Pat.Nr.** | **Examination date** | **MR** | **T** | **TR [ms]** | **TE [ms]** | **FA [°]** | **Matrix** | **Slice thickness [mm2]** | **Number of slices** | **TI [ms]** | **Contrast Agent** | **Contrast Agent dose [ml]** | **Body Weight [kg]** |
| --- | --- | --- | --- | --- | --- | --- | --- | --- | --- | --- | --- | --- | --- |
| 1 | 09.07.2013 | Siemens Trio Tim | 3 | 1800 | 3.79 | 12 | 256x256 | 1 | 190 | 1100 | Gadoteric acid | 10 | 52 |
| 2 | 31.01.2017 | Siemens Trio Tim | 3 | 1800 | 3.79 | 12 | 192x192 | 1 | 190 | 1100 | Gadobutrol | 7.5 | 85 |
| 3 | 21.02.2017 | Siemens Trio Tim | 3 | 1800 | 2.19 | 10 | 256x256 | 1 | 176 | 900 | Gadobutrol | 7.5 | 70 |
| 4 | 19.12.2016 | Siemens Trio Tim | 3 | 1800 | 3.79 | 12 | 256x256 | 1 | 192 | 1100 | Gadobutrol | 10.5 | 63 |
| 5 | 17.02.2010 | Siemens Trio Tim | 3 | 1800 | 3.79 | 12 | 224x256 | 1 | 192 | 1100 | Gadoteric acid | 10 | 65 |
| 6 | 12.01.2016 | Siemens Trio Tim | 3 | 1800 | 2.19 | 10 | 256x256 | 1 | 176 | 1800 | Gadobutrol | 7.5 | 95 |
| 7 | 21.03.2016 | Siemens Skyra* | 3 | 28 | 6 | 27 | 192x256 | 3 | 60 | - | ? | ? | 53 |
| 8 | 09.05.2017 | Siemens Trio Tim | 3 | 1800 | 2.19 | 10 | 484x484 | 1 | 176 | 900 | Gadobutrol | 6 | 60 |
| 9 | 04.03.2014 | Siemens Trio Tim | 3 | 1750 | 3.79 | 12 | 256x256 | 1 | 176 | 1100 | Gadobutrol | 7.5 | 68 |
| 10 | 20.01.2011 | Siemens Trio Tim | 3 | 1540 | 2.19 | 9 | 484x484 | 1 | 160 | 900 | Gadobutrol | 10 | 100 |
| 11 | 21.03.2012 | Siemens Trio Tim | 3 | 1710 | 3.79 | 12 | 256x256 | 1 | 160 | 1100 | Gadobutrol | 7.5 | 53 |
| 12 | 07.03.2017 | Siemens Trio Tim | 3 | 1800 | 2.19 | 10 | 256x256 | 1 | 176 | 900 | Gadoteric acid | 10 | 52 |
| 13 | 03.10.2017 | Siemens Trio Tim | 3 | 1800 | 2.19 | 10 | 256x256 | 1 | 176 | 900 | ? | ? | 62 |
| 14 | 17.08.2017 | Siemens Trio Tim | 3 | 1800 | 3.79 | 12 | 256x256 | 1 | 192 | 1100 | Gadoteric acid | 12 | 66 |
| 15 | 31.05.2016 | Siemens Trio Tim | 3 | 1800 | 2.19 | 10 | 484x484 | 1 | 183 | 900 | Gadobutrol | 9.5 | 95 |

**Supplementary Table 1:**

MR parameters of MS patients with cranial nerve enhancement (CNE). Patient Nr. 7 was examined on an external MR unit, and the contrast agent type and concentration were not known for patients NR.7 and 13. TR (time to repetition), TE (time to echo), FA (flip angle), TI (time to inversion). CNE was detected only at 3T.

| **MS with CNE** | | **MS without CNE Matched for:**  ***Age at MS onset*** | | **MS without CNE Matched for:**  ***Age at MRI*** | |
| --- | --- | --- | --- | --- | --- |
| Pat. Nr. | Number of brainstem  lesions | Pat. Nr. | Number of brainstem  lesions | Pat. Nr. | Number of brainstem  lesions |
| 1 | 3 | 16 | 1 | 31 | 0 |
| 2 | 1 | 17 | 2 | 32 | 0 |
| 3 | 1 | 18 | 1 | 33 | 0 |
| 4 | 4 | 19 | 1 | 34 | 1 |
| 5 | 0 | 20 | 0 | 35 | 1 |
| 6 | 2 | 21 | 1 | 36 | 0 |
| 7 | 0 | 22 | 0 | 37 | 0 |
| 8 | 0 | 23 | 1 | 38 | 0 |
| 9 | 1 | 24 | 0 | 39 | 0 |
| 10 | 0 | 25 | 0 | 40 | 1 |
| 11 | 1 | 26 | 0 | 41 | 0 |
| 12 | 1 | 27 | 0 | 42 | 0 |
| 13 | 0 | 28 | 1 | 43 | 0 |
| 14 | 1 | 29 | 2 | 44 | 0 |
| 15 | 0 | 30 | 0 | 45 | 0 |

**Supplementary Table 2:**

The frequency of T2 lesions in the brainstem (including the mesencephalon, the pons, and the medulla oblongata) were calculated on a patient basis in patients with cranial nerve enhancement (CNE). Patients were matched for age at MS onset 23 (15-29), gender (female male ratio: 8/7), and disease course (SPMS: 2; RRMS: 13). Despite a trend, the number of brainstem lesions (the mesencephalon, the pons and the medulla oblongata) did not differ significantly at the given sample size between cases with (1 (0-2)) and without CNE (1 (0-1)), p< 0.267.

A second set of patients was matched according to age at the time-point of the MRI examination (30 (23-32)), gender (female male ratio: 8/7), and disease course (SPMS: 2; RRMS: 13). Age-matched MS patients had significantly less brainstem lesions than patients with CNE (p=0.041).

Scale variables are provided as median, with a 25%-75% range.

RRMS: relapsing-remitting multiple sclerosis, SPMS: secondary progressive MS

CNE: cranial nerve enhancement

**Supplementary Fig. 1:**

MRIs with suspected cranial nerve enhancement (CNE) based on initial visual assessment were subsequently subjected to region of interest (ROI) based manual measurement of the CN signal intensity (SI) on pre- and post- contrast T1 weighted images with IMPAX EE R20 XV SU3, AGFA Healthcare, Mortsel, Belgium. Freehand regions of interest (ROI) measurements were performed for each cranial nerve with suspected enhancement in pre- and postcontrast images. Each SI measurement was normalized by the SI of the cerebrospinal fluid (CSF) in the lateral ventricles of the same MR sequence. The ratio between the CSF- normalized CN-SIs on pre- and post- contrast images was calculated. CNs with an increase of SI greater than 110% were rated as positive. In these MS patients, the SI of this affected CN was additionally measured in the same manner in a second step in a negatively rated MR scan. This was not possible for two cases, where the affected CN remained enhancing on post contrast T1 weighted images in all available MR scans.

MRI: Magnetic resonance imaging; CNE: Cranial nerve enhancement; SI: signal intensity; ROI: regions of interest, CSF: cerebrospinal fluid; MS: multiple sclerosis.

**Supplementary Fig.2:**

Sup.Fig.2 A-C: (Pat.Nr.4) Coronal reconstructions (A: FLAIR-3D), (B: T1 pre-contrast), (C: T1 post contrast). Pat.Nr.4 presented with a newly detected FLAIR hyperintense right sided medullary lesion involving the nuclear origin of the vagal nerve (Sup.Fig.2A, white arrow). Coronal reconstructions from a T1 weighted image before contrast administration show a black hole in this location (white arrow). Post contrast T1 weighted image revealed no ring enhancement. A short contrast enhancing structure at the level of the right sided vagal REZ was present that extended into the subarachnoid space (Sup.Fig.2C, white arrow), most likely presenting a short segmental extension of the contrast enhancement into the proximal intraparenchymal and subarachnoidal vagal nerve (Sup.Fig.2B white arrow) on the right side. No link of this contrast enhancement to small vessels was present on post- (Sup.Fig.2B) and pre-contrast T1 weighted images (Sup.Fig.2C). Contralateral uvular deviation and ipsilateral palatal drop were noted after initial visual screening for cranial nerve enhancement by retrospectively chard review according to a routine clinical examination that was performed six days after this MRI. Together with the clinical correlate these findings suggest a central right sided vagal involvement in association with an intramedullary and short segment subarachnoidal-peripheral extension in this patient due to a MS plaque in the ambiguous nucleus.
