# Supplementary figures and images for: Cranial Nerve Enhancement in Multiple Sclerosis Is Associated With Younger Age at Onset and More Severe Disease
Source: Front Neurol. 2019 Nov 6;10:1085. doi: 10.3389/fneur.2019.01085 (PMC6851051; doi:10.3389/fneur.2019.01085)

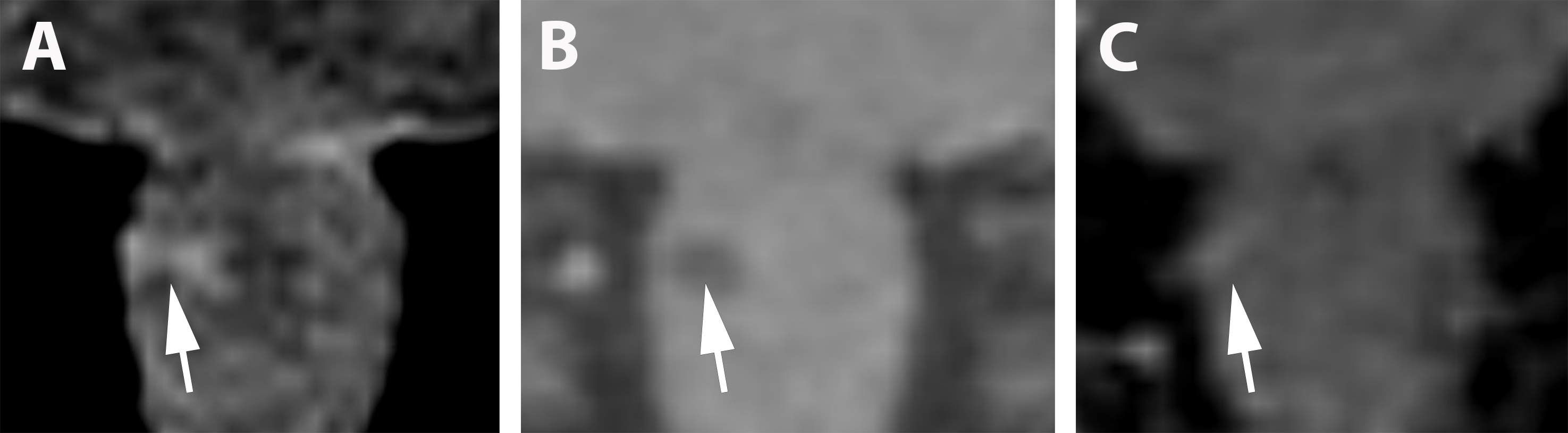

Supplement: Supplementary file 3 [file Image_2.TIF]
